# The Need for Medical Organization in Georgia

**Published:** 1905-03

**Authors:** J. N. McCormack

**Affiliations:** Chairman of the Committee on Organization


### THE NEED FOR MEDICAL ORGANIZATION IN GEORGIA.

BY J. N. McCORMACK, M.D.,
Chairman of the Committee on Organization.

After a pleasant vacation in Cuba and Florida, during which just enough work was done to relieve the monotony, I began to meet the appointments previously made for me by the Georgia State Society. Beginning at Savannah, in the southeastern part of the State, I crossed from city to city to the southwestern side, then up to Columbus and across by Atlanta, Athens and Madison, closing at Augusta on February 2d., and then taking up a similar itinerary in South Carolina.

Aside from the unusual and extreme weather, and the long and often unseasonable hours of travel necessary to keep the engagements, the personal experience was most agreeable. Cordial, old-fashioned hospitality greeted me everywhere, and in the families of the physicians I met some of the finest and most interesting women to be seen in any country.

The condition of the profession of Georgia constantly impressed me as most unique. In one of the great States of the union, well denominated "The Empire State of the South," with its leaders ranking with the best class in this country, there never has been any real, practical attempt at organization. As a result, although in proportion to population there seemed to be less overcrowding of the profession than usual, poverty among its members, with all of the attendant evils to them, and still greater danger to the public, seemed, after careful enquiry, to be more general and pronounced than in any other State I have visited. For instance, although there were notable individual exceptions, a smaller percentage of the physicians than usual owned their own homes, or had ever taken post-graduate instruction, always in my experience fair tests of professional conditions. Several gentlemen courteously called upon me to explain that they were too busy to attend the meetings, or to give the time necessary for membership in county societies, and yet in nearly every case of this kind it developed upon inquiry that these worthy, well-meaning, middle-aged men, most of whom frankly said that they had never attended a medical meeting of any kind since their graduation, not only lived in rented houses, but, what was still more important to them and their patrons, had never been able to avail themselves of the cheap, and, to those not in touch with an active local society, the almost essential post-graduate instruction to be had in every medical center. As a lifetime student of medical sociology these observations were most instructive, each conveying its own lesson and suggesting its own remedy.

The lack of organization was forcibly illustrated in another and larger way. The executive boards, the State Board of Health and State Board of Medical Examiners, the legal arms through which a properly organized profession must make itself effective in all public matters, although made up of good men, are not only subject to political appointment, and tenure, but have no connection or means of co-operation with each other, or with the State Society, a co-operation now recognized as essential between all of the bodies managing professional interests in any State. This means, of course, that such boards are practically deprived of the moral support of their medical constituency in their every-day work, that quackery in all its most flagrant forms is rampant, with the press and public sentiment on its side, while a profession capable of great strength, if organized and united, is helpless to secure or to enforce remedial legislation.

The State Society, on the other hand, and this will be a surprise to those of the old regime who have given undue importance to large central societies, State, inter-State or national, as factors in organization work, is in a flourishing condition, largely owing to the efforts of its most efficient secretary. It has a membership of seven hundred, out of a total eligible medical population of three thousand, always has a fair attendance and an interesting program, and is in every way fully up to the average of bodies of this kind under the old order of things. That such a large, worthy and long-established organization has exerted so little influence upon either the profession or public will be readily understood when it is known that it has no real foundation, having no local societies in affiliation with it, and that no systematic attempt has ever been made to foster these or to reach and benefit the rank and file of the profession, that large and important element everywhere which can not hope to attend State meetings regularly, but is none the less entitled to representation and a voice. Besides, they have practically but a two-days' session, meeting usually about noon the first day and adjourning at the same time on the third, and the short business session are really mass-meetings of those present, who do not claim to represent anybody but themselves, almost of necessity limited to the transaction of routine business, with neither the time nor opportunity for considering the real needs of the profession and State in the broad way which their importance demands.

All of this seems the more remarkable in a State divided only by an arbitrary line from Alabama, where for more than a generation every county, some of them containing but three or four physicians, has had an effective county society in full legal control of its medical and health affairs, with over ninety-two per cent. of the entire profession enrolled as permanent members of both these and the State association, and with its organized profession made by law as much a part of the warp and woof of the county and State governments as the legislative, judicial and executive departments. As has been more recently demonstrated in many other States, the organization has been so effective in Alabama not so much on account of its strong State association, as because it is rooted and grounded in the body of the profession in every section, and that it exists, and unselfishly exercises the great powers which years of harmony and co-operation have given it, for the benefit not only of its own members, but of the public also, whose interests are inseparable from ours.

As the question of reorganization is now pending before the State Society, I did not deem it proper for me to discuss this phase of the subject in any of my talks, confining myself to an exposition of the methods, purposes and possibilities of local or county societies as the only feasible foundation for such a far-reaching, comprehensive organization of the profession of this country as is demanded to minimize the evils which have so long dominated it, and give it that voice and influence in the control of all affairs within its realm of which it has heretofore been deprived by dissensions within its own ranks. I explained and urged that these dissensions had never existed except between local men, those who think they have competing interests, and that they can only be minimized and finally cured, as has now been abundantly demonstrated, by such systematic, all-pervading local organizations as will bring these worthy men together in frequent and unrestricted consultation over the condition and needs of the profession and people of their jurisdiction. I insisted that the American Medical Association, aside from the scientific work done in the sections during four days of the year, was only an essential factor in this work as a bond of union between the States, for concentrating and making effective the instructions coming to it through the delegates from the States, and for the maintenance and distribution of its great Journal; that the State Societies, in session but two or three days, in addition to the scientific work done, were only important as a bond of union between the counties, as a help in promoting organization in these, and for voicing and putting into operation the instructions of the delegates from its constituent bodies; but that the county societies, which must be the foundation and groundwork of any real organization, could meet monthly, or even weekly, as many of them are doing, not only for scientific and modern postgraduate work, but for the cultivation of such personal relations as will make the profession a credit to itself and the community, and ultimately unite it in the study and promotion in every interest in which it is concerned. I advised them frankly that this would be difficult work at the outset, that no plan which could be proposed for such local societies would be found either perfect or self-operating, that local medical organization is a tree rather than a vine, and that the Alabama plan, simplified and made more democratic by the Association, was intended only as a trellis or framework for the assistance and support of such organizations during the evolutionary period of their growth.

While I did not feel at liberty to talk upon the subject of State reorganization in my addresses, in the discussion which nearly always followed, and in private conversation, no such restriction could be imposed, and others expressed themselves freely, as they had a right to do. Inquiries were made as to every detail of the methods suggested for local improvement, and the interest and enthusiasm were almost universal. In fact, the only discordant note I heard was from a little journal whose editor was expelled from the State Society at its last meeting after I had, without his knowledge, urged a milder sentence, and who naturally feels sore at everything in the way of medical organization. When it is known that the proposed constitution expressly provides that only white physicians are eligible for membership, and that affiliation with the American Medical Association is entirely voluntary, and can be severed by Georgia or any other State the first day it becomes undesirable for any reason, and that the Association can have no possible interest in the profession of any State except for the mutual help to come from such relations, his criticisms, in so far as they had any weight, only served to strengthen the position of those who want to put Georgia in the ranks of organized medicine.

Under a proper system of organization the possibilities before this profession are almost beyond the conception of its members, so long has the State been under the almost complete domination of the quack interests. Not an obstacle suggested, in the way of small and sparsely settled counties, difficulties in travel, and other like things, but have been met and overcome in Texas, Arkansas Michigan, Kentucky and other States, which have already tried this plan with such good results. Experience has demonstrated, over and over again, that even the fear that a single member of the present State Society might be lost is groundless. As there are now practically no county societies such members would return home and become the natural centers around which these would be formed, and the State Society could easily have two thousand members by the next annual meeting. In one year Texas increased the membership from 390 to 2,510, Michigan from 452 to 2,100, and so on through the list of States. A percentage equal to Alabama would give Georgia 2,760 members, but this could only be hoped for after several years of painstaking work. With such prospects possible under the plan proposed it is submitted to this great profession that no one should raise his voice in opposition unless he has something better to offer.

